# Apocrine mixed tumor of the eyelid: a case report

**DOI:** 10.1186/s13000-016-0483-5

**Published:** 2016-03-24

**Authors:** Aileen Azari-Yam, Mohammad Abrishami

**Affiliations:** Department of Medical Genetics, School of Medicine, Tehran University of Medical Sciences, Tehran, Iran; Department of Molecular Medicine, Biotechnology Research Center, Pasteur Institute of Iran, Tehran, Iran; Negah Eye Center, Tehran, Iran

## Abstract

**Background:**

Apocrine mixed tumor is usually found in parotid glands. Its cutaneous counterpart is rare and its occurrence in the eyelids is even rarer.

**Case presentation:**

This study reports an apocrine mixed tumor of the upper left eyelid in a 68 year-old lady with a history of breast cancer. This mass appeared about 3 years ago, as a slowly growing small nodule, and was completely excised. On microscopic examination, an encapsulated mass with epithelial and mesenchymal features was seen. The epithelial component presented tubular, cystic and infundibular structures while the mesenchymal component was fibrous in some areas and myxoid in others. Plasmacytoid hyaline cells, lipomatous change and focal calcification were appreciated focally. Immunohistochemical studies showed stromal staining for CD10, S-100, α-SMA and p63. Luminal cell layer of the epithelial component was positive for EMA, CK-7 and GCDFP-15 markers. The capsule was unbreached and no satellite lesions were appreciated. No evidence of relapse was evident after 16 months of follow-up.

**Conclusions:**

The diagnosis of eyelid tumors of adnexal origin can be challenging because they are rare and display a wide range of morphological patterns, as the tumor cells might differentiate along any line of the folliculosebaceous-apocrine system. Immunohistochemistry helps improve the accuracy of assessment.

## Background

Pleomorphic adenoma or benign mixed tumor is a dual cell neoplasia, which is the reason for the designation of this tumor as mixed. The first component is of epithelial (apocrine or eccrine) origin and morphologically shows various arrangements including ductal, cystic and solid while also showing structures with a double-layer of inner cuboidal and outer myoepithelial cells. Immunohistochemical study of myoepithelial markers reveals the immunologic nature of the outer cell layer. The second component is of mesenchymal origin and shows fibrous, myxoid, mucoid, and chondroid features. The mesenchymal component is also very diverse and reveals mucoid, myxoid, chondroid, osseous, and adipose features. Mixed tumor is usually found in parotid glands. Cutaneous pleomorphic adenoma is rare and its occurrence in the eyelids is even rarer. This tumor is of variants; eccrine and apocrine tumors. The latter is identified by its morphological features and immunohistochemistry helps to establish its diagnosis. It is not always possible to specify the exact origin of the tumor. Complete surgical removal is the treatment of choice.

## Case presentation

A 68 year-old woman with a history (12 years ago) of breast cancer presented with a 0.7 cm × 0.7 cm solitary nodule of the left upper eyelid. The mass appeared three years ago in the central lid, was slowly growing and the patient reported no pain. On physical examination, the mass was firm, freely moving and not tender. The overlying skin did not reveal any abnormality. The clinical diagnosis was epidermal inclusion cyst and the mass was removed for diagnostic as well as cosmetic reasons.

The specimen was received in 10 % buffered formalin. On gross inspection, a tan well-circumscribed dermal mass measuring 0.7 cm × 0.7 cm × 0.5 cm was seen. The overlying skin ellipse (0.8 cm × 0.4 cm) was grossly unremarkable with a rubbery and firm consistency. On the cut surface, it was apparently homogenous. There was no attachment to underlying structures. On sectioning, no gross keratinous or cheesy material was seen.

**Fig. 1:**
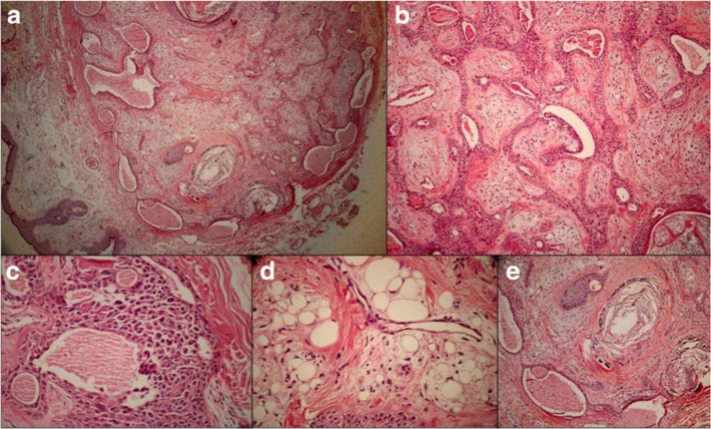
Histomorphological features of the apocrine mixed tumor. **a** Low power view of the tumor with unbreached fibrous capsule and intact epidermis at the left lower corner. Dual epithelial and mesenchymal components are seen (hematoxylin and eosin × 40). **b** Bilayer arrangement and snouting of the epithelial cells around the ducts and cystic structures (hematoxylin and eosin × 100). **c** Plasmacytoid differentiation of tumor cells or hyaline cells (hematoxylin and eosin × 400). **d** Lipomatous differentiation (hematoxylin and eosin × 400). **e** Cystic structures with lamellar keratinized material, calcification and histiocytic giant cells (hematoxylin and eosin × 40)

Hematoxillin and eosin staining of the sections revealed a dermal mass with fibrous encapsulation (Fig. 1a). The tumor consisted of dual epithelial and stromal components. The former consisted of interconnected cords, tubules, solid islands and cystic structures showing a two layer arrangement of cuboidal epithelial cells (Fig. 1b). The tubular structures showed decapitation secretion or apical snouting of the inner cell layer. Keratotic lamella-filled cysts lined with squamous epithelium were seen. The latter contained anucleated squamous cells. As a result of the eccentrically placed nuclei, hyaline epithelial cells were observed focally to have a plasmacytoid appearance (Fig. 1c). The stroma was myxoid with intersecting collagen fibers, focal lipomatous differentiation (Fig. 1d) and calcification (Fig. 1e). Capsular breach, hypercellularity, atypia, high mitotic figures or satellite lesions were not appreciated. Microscopically, the tumor had no connection to the palpebral lobe of the lacrimal gland.

### Immunohistochemistry

**Fig. 2:**
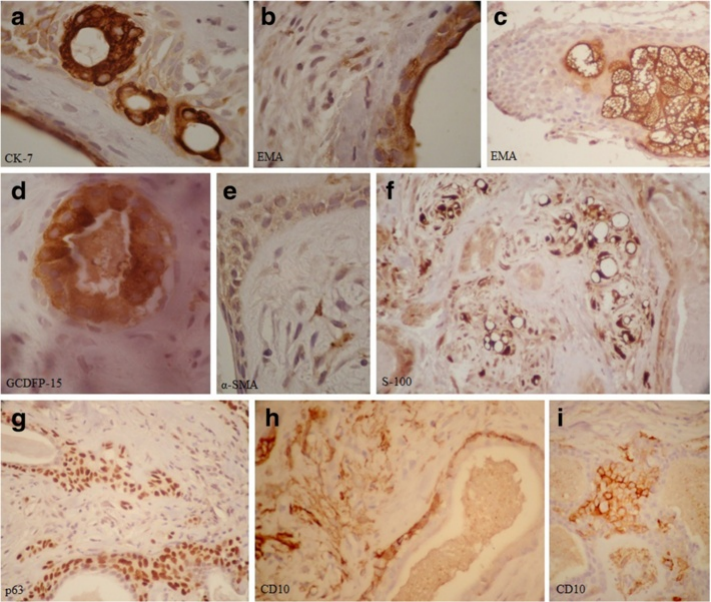
Representative photomicrographs of immunophenotyping in apocrine mixed tumor of the eyelid. **a** Strong positive CK-7 staining of the ductal luminal cells. The abluminal and stromal cells are negative. **b** Apical staining of luminal cells with epithelial membrane antigen (EMA) marker. **c** Strong bubbly EMA positive sebaceous cells of the overlying non-tumoral skin. **d** Strong luminal cell reactivity with gross cystic disease fluid protein-15 (GCDFP-15). **e** Negative epithelial component with α-SMA (top and left) and focal positive stromal cells. **f** S-100 strong positivity of lipomatous cells of the stroma and negative epithelial component (right and bottom left corner). **g** P63 shows positive abluminal cells of the ductal structure with dispersion or streaming of the myoepithelial components into stroma. The luminal cell layer is negative. **h** Stromal and focal abluminal CD10 reactivity. **i** Staining of plasmacytoid cells with CD10 marker. Original magnifications: ×400 for (**a**), (**b**), (**d**) and (**e**); ×1000 for (**c**), (**f**), (**g**), (**h**), and (**i**)

To better characterize the cell nature, differentiation, and protein expression of this tumor, we performed an extended immunohistochemistry panel for CK7 (cytokeratin-7-7), EMA (epithelial membrane antigen), GCDFP-15 (gross cystic disease fluid protein-15), α-SMA (alpha- smooth muscle actin), S-100, p63, and CD10 immunomarkers (Novocastra Laboratories; Leica Microsystems). Both internal and external positive and negative controls were assessed according to personal experience and manufacturer instructions. CK-7 immunostaining was positive at the inner cell layer of the ducts, tubules and cystic structures (luminal cells). The abluminal (basal/myoepithelial) cells and stromal component were not reactive (Fig. 2a). EMA which is present in a variety of glandular (secretory) epithelia such as breast, eccrine and apocrine glands was positive in the luminal cells of the epithelial component and displayed an apical staining pattern (Fig. 2b); the sebaceous cells were bubbly positive (Fig. 2c). Some non-specific epidermal staining was observed. GCDFP-15 immunostaining showed strong luminal positivity (Fig. 2d). The abluminal and stromal cells were negative. Α-SMA reacts with tumors arising from smooth muscles and myoepithelial cells and was negative at epithelial component, but showed focal positive stromal staining (Fig. 2e). The vascular walls within the tumor showed strong cytoplasmic reactivity. S-100 immunostaining revealed strong stromal positivity, especially in the lipomatous areas (Fig. 2f). The epithelial component was not reactive. The p63 immunostaining showed positivity at the abluminal cells of tubules and cystic structures. The stromal cells were focally positive (Fig. 2g). CD10, or CALLA (common acute lymphoblastic leukemia antigen) is a cell surface antigen that has been confirmed as a myoepithelial cell marker. CD10 immunostaining showed reactivity in abluminal cells and stromal fibrocytes (Fig. 2h). The plasmacytoid cells were also positive for this marker (Fig. 2i).

## Discussion

A rare case of apocrine mixed tumor of the eyelid has been reported in a 68-year old lady with a history of breast cancer. A metastatic process was not considered clinically for the eyelid tumor because of its slow growth rate and mobile nature. Apocrine mixed tumors most commonly occur in the salivary glands but are also found in other sites. To the best of our knowledge, only 25 tumors of this type have been reported in the eyelid [1–12]. In the periorbital area, the most common site of origin is the lacrimal gland (Krause’s glands) [13, 14]. Sweat glands, have more rarely been reported as the site of origin [15]. In some cases, the exact origin of the tumor could be determined by thorough clinical examination. Correlation of physical signs and a thorough search for any remnants of the possibly affected lacrimal gland lobe might help to determine the tumor origin. Our case showed no attachment to the lacrimal gland.

The hematoxiline eosin staining is the gold standard for diagnosis of tumors arising in skin adnexa and IHC is considered supplementary. Some studies have emphasized the importance of immunohistochemistry in the diagnosis of mixed tumors [16]. To better characterize cell nature, differentiation, and protein expression of this tumor at this rare site, we performed an extended immunohistochemistry panel. Immunohistochemical analysis showed stromal staining for CD10, S-100, α-SMA and p63. The luminal cell layer of the epithelial component was positive for EMA, CK-7 and GCDFP-15 markers. These findings are consistent with the biphasic nature of this tumor. Similar findings have been reported in previous studies [17, 18].

Immunostaining with myoepithelial markers for S-100, p63, and CD10 is also of benefit for detection of reminiscent benign myoepithelial cells in cases of carcinoma ex pleomorphic adenoma [19].

An interesting feature of our case is that the tumor displays a wide range of differentiation and metaplastic changes in its epithelial, myoepithelial and stromal components. The spectrum of metaplastic changes included changes in the myoepithelial component documented as hyaline or plasmacytoid cells, spindling, and collagenous spherulosis. Stromal alterations included adipose metaplasia and calcification.

The histomorphological differential diagnosis includes eccrine and apocrine hidrocystomas, fibroadenoma, hidradenoma, mucinous adenocarcinoma, adenoid cystic carcinoma and rare soft tissue tumors like the myxoid chondrosarcoma. Differentiation from hidrocystomas was mostly based on clinical grounds as apocrine hidrocystomas most commonly appear as translucent papules or nodules. Other differentials are ruled out mostly on histomorphological basis. The tumor in this study presented hyaline cells which are usually absent in other types of adnexal tumors [16]. Cellular atypia and pleomorphism were absent in our tumor, a feature which differentiates it from malignant entities.

By morphologic features, the apocrine versus eccrine origin of the tumor can be inferred. Apocrine glands are restricted to the axilla, nipples, genital and anal areas. In the periocular area, they emerge as glands of Moll. The most characteristic differentiating feature which favors an apocrine origin is the double layer arrangement of the branching tubular structures. It is widely accepted that the outer epithelial layer of these structures is of myoepithelial origin confirmed by immunophenotypic studies. Another feature favoring apocrine origin is the scalloping of the glands towards the luminae or decapitation secretion. In our case, both features were present.

The benign versus malignant nature of this tumor could be inferred by some clinical and histomorphological features. Despite their innocent morphological appearance, it is widely accepted that mixed tumors could be infiltrative or malignant. The most reliable guide to establishing the malignant nature of an adnexal tumor is the demonstration of an infiltrative margin or aggression to the capsule. High mitoses counts have not been agreed upon as a factor of recurrence or metastasis. Hypercellularity, atypia and pleomorphism have been mentioned as factors to classify the tumor as potentially malignant. In the case of the tumor reported here, an unbreached encapsulation was evident. Hypercellularity, atypia, high mitotic figures or satellite lesions were not appreciated. Focal calcification was seen in our tumor (Fig. 1d) however, it is not a malignancy indicator [17].

Some studies have emphasized the importance of immunohistochemistry, in the diagnosis of mixed tumors [18].

The diagnosis of eyelid tumors of adnexal origin can be challenging because they display a wide range of morphological patterns, as the tumor cells might differentiate along any line of the folliculosebaceous-apocrine system.

## Conclusion

Apocrine mixed tumor can arise from adnexal structures of the eyelid and has dual (myo) epithelial and mesenchymal components. Its encapsulated margin and bland cytological features help establish it as a benign lesion. In clinical practice, the histopathological diagnosis of adnexal tumors is made carefully through the assessment of the growth pattern of the histological architecture, cellular differentiation, and features of the tumor stroma, along with the clinical information. Immunohistochemistry helps improve the accuracy of assessment. Complete surgical excision is the treatment of choice.

### Consent

Written informed consent was obtained from the patient for publication of this Case Report and any accompanying images. A copy of the written consent is available for review by the Editor-in-Chief of this journal.

## Abbreviations

CK7: Cytokeratin-7
CALLA: common acute lymphoblastic leukemia antigen
EMA: epithelial membrane antigen
GCDFP-15: gross cystic disease fluid protein-15
α-SMA: alpha- smooth muscle actin.
